# Negative Effects of Mental Fatigue on Performance in the Yo-Yo Test, Loughborough Soccer Passing and Shooting Tests: A Meta-Analysis

**DOI:** 10.3390/jfmk7010010

**Published:** 2022-01-13

**Authors:** Jozo Grgic, Ivan Mikulic, Pavle Mikulic

**Affiliations:** 1 Institute for Health and Sport, Victoria University, Melbourne, VIC 3011, Australia; 2 Faculty of Kinesiology, University of Zagreb, 10000 Zagreb, Croatia; ivan.mikulic@kif.unizg.hr (I.M.); pavle.mikulic@kif.unizg.hr (P.M.)

**Keywords:** data synthesis, cognitive task, mental fatigue, performance, soccer, team-sport

## Abstract

We aimed to examine the effects of mental fatigue on the Yo-Yo test and Loughborough soccer passing and shooting tests performance using a meta-analysis. The search for studies was performed through eight bibliographic databases (Academic Search Elite, AUSPORT, Cochrane Library, PsycInfo, PubMed/MEDLINE, Scopus, SPORTDiscus, and Web of Science). The methodological quality of the included studies was assessed using the PEDro checklist. A random-effects meta-analysis was performed for data analysis. After reviewing 599 search results, seven studies with a total of ten groups were included in the review. All studies were classified as being of good methodological quality. Mental fatigue reduced the distance covered in the Yo-Yo test (Cohen’s *d*: −0.49; 95% confidence interval [CI]: −0.66, −0.32). In the Loughborough soccer passing test, mental fatigue increased the original time needed to complete the test (Cohen’s *d*: −0.24; 95% CI: −0.46, −0.03), increased penalty time (Cohen’s *d*: −0.39; 95% CI: −0.46, −0.31), and decreased performance time (Cohen’s *d*: −0.52; 95% CI: −0.80, −0.24). In the Loughborough soccer shooting test, mental fatigue decreased points per shot (Cohen’s *d*: −0.37; 95% CI: −0.70, −0.04) and shot speed (Cohen’s *d*: −0.35; 95% CI: −0.64, −0.06). Overall, the findings presented in this review demonstrated that mental fatigue negatively impacts endurance-based running performance as well as soccer passing and shooting skills.

## 1. Introduction

Mental fatigue is commonly defined as “a psychobiological state caused by prolonged exertion that has the potential to reduce cognitive performance and exercise performance” [1]. Researchers have explored the effects of mental fatigue on performance in various modes of exercise [1]. A seminal study by Marcora et al. [2] reported that mental fatigue induced by a demanding cognitive task negatively impacted performance in cycling to exhaustion at 80% of peak power output. Since this study, research has also examined the effects of mental fatigue on various sport-specific outcomes [3].

For example, in soccer, studies have explored the effects of mental fatigue on performance in the Yo-Yo test, Loughborough soccer passing test, and Loughborough soccer shooting test [4,5,6,7,8,9,10]. Briefly, the Yo-Yo intermittent recovery test is a popular test used to evaluate performance in interval running [11,12,13]. This test determines an individual’s capacity to undertake, repeatedly perform, and recover from high-intensity running [11,12,13]. Due to its structure, the Yo-Yo test is particularly relevant for team sports [11]. While the Yo-Yo test examines endurance performance, the Loughborough soccer passing test and Loughborough soccer shooting test evaluate sport-specific skill levels [14,15] (i.e., passing, controlling, and shooting the ball; Table 1).

Studies evaluated the effects of mental fatigue on performance in these tests, but the findings varied. For example, the mental fatigue-induced reduction in Yo-Yo test performance ranged from small (Cohen’s *d*: −0.21) to very large (Cohen’s *d*: −1.34), making it difficult to establish the true effect in the population [4,5]. In the Loughborough soccer passing test, some studies have reported that mental fatigue negatively impacts performance time, whereas others did not find a significant difference between the control and mental fatigue trials [4,5]. A similar discrepancy has been observed for outcomes such as shot speed in the Loughborough soccer shooting test [4,8].

Several reviews have been published that summarized the effects of mental fatigue on exercise performance [3,16,17]. However, these reviews have not specifically focused on the Yo-Yo test and Loughborough soccer passing or shooting tests. Perhaps more importantly, these reviews did not contain a meta-analysis that pooled the data from all studies on the topic. This would be highly relevant to perform given that some of these studies might have been underpowered to find significant differences. Accordingly, this review aimed to perform a meta-analysis examining the effects of mental fatigue on performance in the Yo-Yo test and Loughborough soccer passing and shooting tests. We hypothesized that mental fatigue would negatively influence performance in these tests.

## 2. Materials and Methods

### 2.1. Literature Search Strategy

The search for studies that explored the effects of mental fatigue on performance in the Yo-Yo test, Loughborough soccer passing test, and/or Loughborough soccer shooting test was carried out in two phases. In the first phase, we performed a search through eight different bibliographic databases, including: Academic Search Elite, AUSPORT, Cochrane Library, PsycInfo, PubMed/MEDLINE, Scopus, SPORTDiscus, and Web of Science. In all of these databases, the following search syntax was applied: (“mental fatigue” OR “mentally fatigued”) AND (“yo-yo” OR “yoyo” OR “yo yo” OR “intermittent endurance” OR “intermittent recovery” OR “Loughborough soccer passing” OR “Loughborough soccer shooting”). After this phase was completed on 5 September 2021, we then screened the reference list from all studies that were found to satisfy the inclusion criteria. Post reference screening, forward citation tracking (i.e., examining the papers that cited the included studies) using the Google Scholar database was conducted.

### 2.2. Selection Criteria

Studies were included in the review if they satisfied the following criteria: (1) examined the effects of mental fatigue on performance in the Yo-Yo test, Loughborough soccer passing test, and/or Loughborough soccer shooting test; (2) used a crossover study design that involved a control trial and a mental fatigue trial; and (3) included humans as study participants.

### 2.3. Data Extraction

We extracted the following data from the included studies: (1) year of study publication and lead author name; (2) participants characteristics (e.g., sex, training status); (3) description of the control and cognitive tasks; (4) performance test and its outcomes; and (5) mean ± standard deviation for the test outcome(s) from the control and mental fatigue trials. One study did not report these data in the manuscript. For this study [4], on request, the data were received from the corresponding author. Two studies [6,7] presented the data needed for the meta-analysis only in figures. For these two studies, the necessary data were extracted using the Web Plot Digitizer software (https://apps.automeris.io/wpd/) (accessed on 10 September 2021).

### 2.4. Quality Assessment

The methodological quality of the included studies was assessed using the validated 11-item PEDro checklist [18]. The items on the PEDro checklist evaluate different methodological aspects, including inclusion criteria, randomization, allocation concealment, blinding of participants and assessors, attrition, and data reporting. All items of the PEDro checklist are scored as “1” (criterion is satisfied) or “0” (criterion is not satisfied). The first item is not included in the total score. Therefore, the maximum possible number of points that can be scored is 10. In accordance with previous reviews, the studies were classified studies as poor, fair, good, or excellent quality if they scored ≤3 points, 4–5 points, 6–8 points, and 9–10 points, respectively [19,20].

### 2.5. Statistical Analysis

The meta-analysis was performed using effect sizes (Cohen’s *d*). Cohen’s *d* effect sizes were calculated using the mean ± standard deviation data from the control and mental fatigue trials, sample size, and correlation between the trials. Correlation between the trials was not reported in any of the included studies. Therefore, correlation values were estimated using the methodological approach recommended in the Cochrane Handbook [21]. A total of seven meta-analyses were performed, for: distance covered in the Yo-Yo test; time needed to complete the test, penalty time, and performance time in the Loughborough soccer passing test; points per shot, shot speed, and shot sequence time in the Loughborough soccer shooting test. The interpretation of effect sizes was based on the following thresholds: trivial (<0.20), small (0.20–0.49), medium (0.50–0.79), and large (≥0.80) [22]. Negative Cohen’s *d* values denote a decrease in performance with mental fatigue. Meta-analyses were performed using the random-effects model [23]. *I*^2^ statistic was used to evaluate heterogeneity. *I*^2^ values were interpreted as low (<50%), moderate (50–75%), and high heterogeneity (>75%). The statistical significance threshold was set at *p* < 0.05. All analyses were performed using the Comprehensive Meta-Analysis software, version 2 (Biostat Inc., Englewood, NJ, USA).

## 3. Results

### 3.1. Search Results

**Figure 1:**
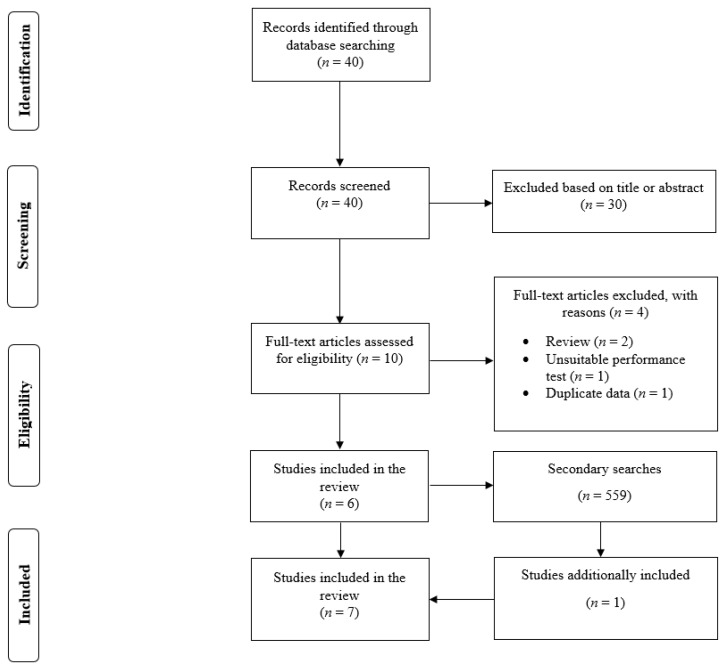
Flow diagram of the search process.

There were 40, 243, and 316 search results in the database search, screening of the reference list, and forward citation tracking phases, respectively (Figure 1). Of the search results found in the databases, ten full-text papers were read and six studies were included [4,5,7,8,9,10]. One additional study [6] was found in the forward citation tracking. Therefore, this review included a total of seven studies [4,5,6,7,8,9,10]. However, one study [4] included three independent groups (i.e., players from under-14, under-16, and under-18). Thus, there was a total of ten groups in the seven included studies.

### 3.2. Summary of Studies

All seven studies explored the effects of mental fatigue on Yo-Yo test performance (10 study groups; Table 2). In all of these studies, the Intermittent Recovery Test Level 1 variant of the Yo-Yo test was used. The pooled number of participants for studies that used the Yo-Yo test was 134. Three studies (5 study groups; *n* = 58) and two studies (4 study groups; *n* = 50) explored the effects of mental fatigue on Loughborough soccer passing test and Loughborough soccer shooting test, respectively. The cognitive task used to induce mental fatigue was the Stroop test in six studies, while one study [5] employed the “Brain It On” application on a smartphone. The duration of the cognitive task was 30 min in all studies. For the control trials, studies used reading of magazines, watching an emotionally neutral video, or no activity. The duration of the control trials for studies that used reading of magazines or watching a video was from 15 to 30 min. Five [4,6,7,8,9] studies explored the effects of the cognitive task on mental fatigue. In all these studies, mental fatigue was higher following the cognitive task.

### 3.3. Methodological Quality

Five studies [5,6,7,9,10] scored six points on the PEDro checklist, whereas two studies [4,8] scored eight points. Therefore, all included studies were classified as being of good methodological quality (Table 3).

### 3.4. Meta-Analysis Results

**Figure 2:**
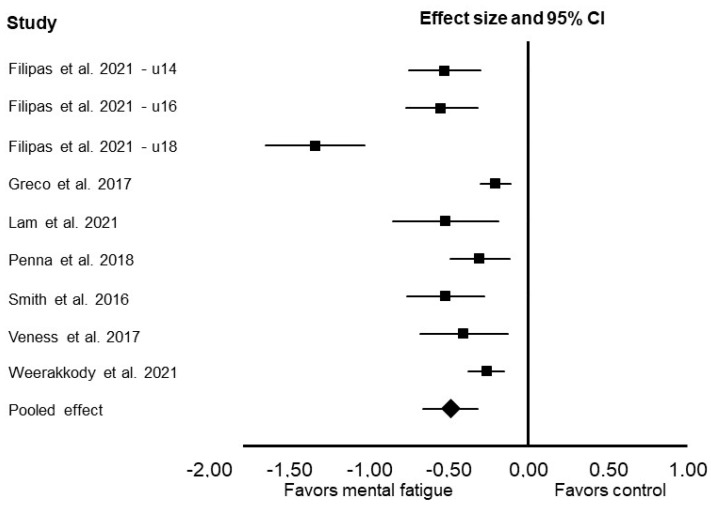
Forest plot presenting the results of the random-effects meta-analysis comparing the effects of control vs. mental fatigue on Yo-Yo test performance. Data are reported as Cohen’s *d* (effect size) and 95% confidence interval (CI). The diamond at the bottom presents the overall effect. The plotted squares denote effect sizes, and the whiskers denote their 95% CIs.

Mental fatigue reduced the distance covered in the Yo-Yo test (Cohen’s *d*: −0.49; 95% confidence interval [CI]: −0.66, −0.32; *p* < 0.001; *I*^2^ = 33%; Figure 2).

**Figure 3:**
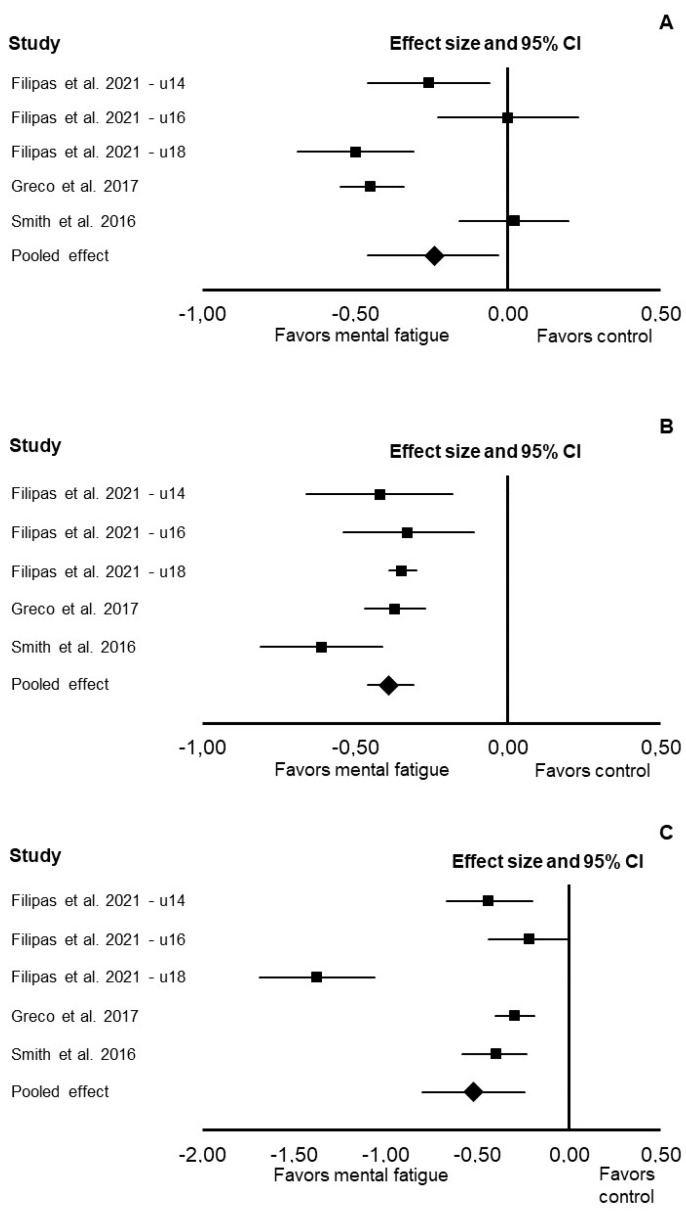
Forest plot presenting the results of the random-effects meta-analysis comparing the effects of control vs. mental fatigue on the original time needed to complete the test (**A**), penalty time (**B**), and performance time (**C**) in the Loughborough soccer passing test. Data are reported as Cohen’s *d* (effect size) and 95% confidence interval (CI). The diamond at the bottom presents the overall effect. The plotted squares denote effect sizes, and the whiskers denote their 95% CIs.

In the Loughborough soccer passing test, mental fatigue increased the original time needed to complete the test (Cohen’s *d*: −0.24; 95% CI: −0.46, −0.03; *p* = 0.024; *I*^2^ = 0%; Figure 3), increased penalty time (Cohen’s *d*: −0.39; 95% CI: −0.46, −0.31; *p* < 0.001; *I*^2^ = 15%), and decreased performance time (Cohen’s *d*: −0.52; 95% CI: −0.80, −0.24; *p* < 0.001; *I*^2^ = 51%).

**Figure 4:**
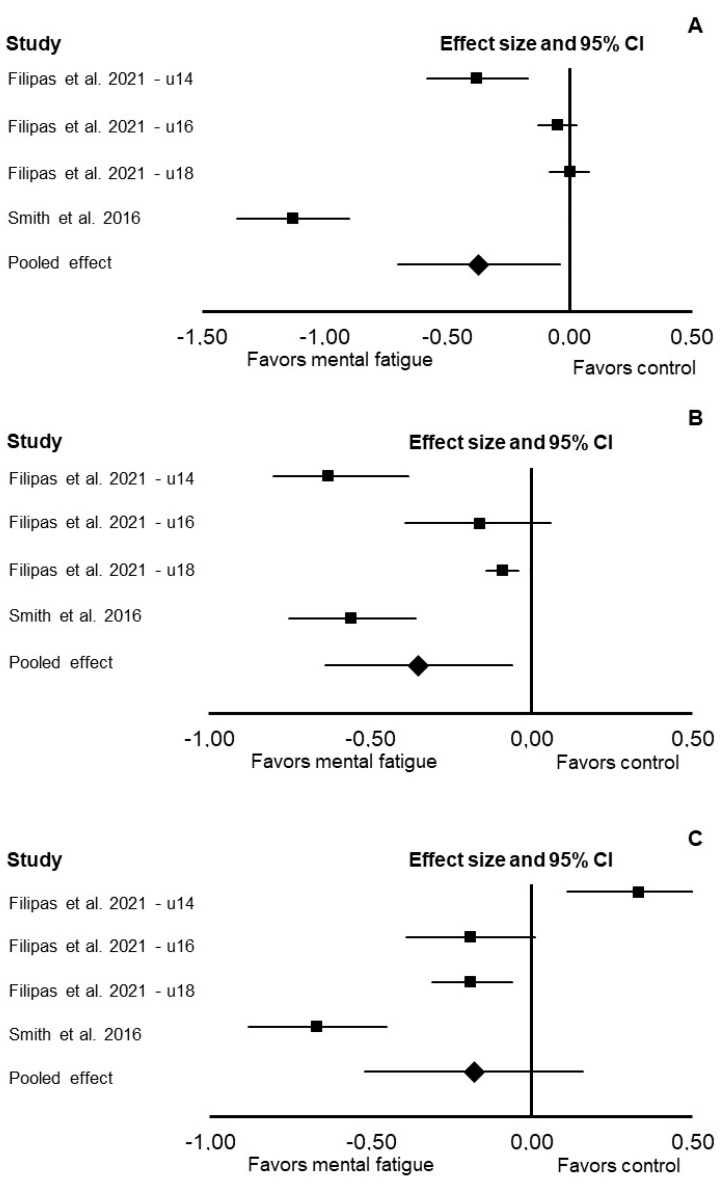
Forest plot presenting the results of the random-effects meta-analysis comparing the effects of control vs. mental fatigue on points per shot (**A**), shot speed (**B**), and shot sequence time (**C**) in the Loughborough soccer shooting test. Data are reported as Cohen’s *d* (effect size) and 95% confidence interval (CI). The diamond at the bottom presents the overall effect. The plotted squares denote effect sizes, and the whiskers denote their 95% CIs.

In the Loughborough soccer shooting test, mental fatigue decreased points per shot (Cohen’s *d*: −0.37; 95% CI: −0.70, −0.04; *p* = 0.028; *I*^2^ = 57%; Figure 4) and shot speed (Cohen’s *d*: −0.35; 95% CI: −0.64, −0.06; *p* = 0.019; *I*^2^ = 0%). There was no significant difference between the control and mental fatigue conditions for shot sequence time (Cohen’s *d*: −0.18; 95% CI: −0.52, 0.16; *p* = 0.300; *I*^2^ = 26%).

## 4. Discussion

In line with our hypothesis, we found that mental fatigue has a negative effect on some aspects of physical and technical exercise performance. Specifically, the distance covered in the Yo-Yo test was lower following the mental fatigue trials. Additionally, it was found that mental fatigue hampered performance in the Loughborough soccer passing test by increasing the original time needed to complete the test and penalty time. As a result, performance time—calculated as original time plus penalty time—was also slower post mental fatigue. Similar to these findings, mental fatigue decreased points per shot and shot speed in the Loughborough soccer shooting test. Overall, these findings demonstrated that mental fatigue negatively impacts endurance-based running performance as well as soccer passing and shooting skills. Most effect sizes were in the range from small to medium and are likely to be practically relevant. These findings are novel, given that previous research on the topic produced conflicting results (i.e., varying effect sizes or differences in findings) [4,5,6,7,8,9,10].

Our results add to the body of evidence demonstrating that mental fatigue may hinder endurance-based exercise performance [3,16,17]. Variables such as heart rate, metabolite accumulation, and neuromuscular function are generally not affected by mental fatigue [24]. Therefore, it has been suggested that the negative effect of mental fatigue on exercise performance is due to the increase in the rating of perceived exertion (RPE). However, some studies evaluated RPE values and reported no significant difference between the control and mental fatigue [5,10]. Still, these studies evaluated RPE at test exhaustion, which needs to be considered given the findings that mentally fatigued athletes display higher RPE values during the Yo-Yo test, but not necessarily at the end of the test [4]. Furthermore, there are nuances to mention in the interpretation even if there is no significant difference in RPE values between the trials at test exhaustion. Specifically, despite no significant RPE differences, it should be considered that the total distance covered in the Yo-Yo test in these studies [5,10] was higher in the control condition, showing that participants who were mentally fatigued covered less distance to achieve a similar level of RPE. It has been suggested that the increase in perceived exertion is mediated by cerebral adenosine accumulation during cognitive tasks [24,25]. Indeed, this hypothesis is supported by data indicating that caffeine supplementation—which acts by binding to adenosine receptors—averts the negative effects of mental fatigue on exercise performance [26,27].

The Yo-Yo test has been reported to correlate with several sport-specific outcomes. For example, a positive correlation (*r* = 0.70–0.81) was found between the distance covered in the Yo-Yo test and the amount of high-intensity running performed during the whole soccer game, during a 5-min period involving peak running intensity, or at the end of each half of a game [11,28,29]. Therefore, it seems likely that the negative effects of mental fatigue on performance in the Yo-Yo test may also translate to sport-specific performance. However, it should be considered that running-based performance in soccer matches is also influenced by a multitude of factors, such as match location, quality of opposition, and match outcome [30]. Therefore, the results obtained during testing may not necessarily be generalized to performance in sports competitions.

Given that the Yo-Yo test is most commonly used for testing (rather than training) purposes, our results highlight that researchers and practitioners should attempt to standardize the cognitive activity before this assessment, particularly when exploring differences between individuals. As demonstrated herein, the negative effect of mental fatigue on performance in the Yo-Yo test may range from small to medium (Cohen’s *d*: −0.49; 95% CI: −0.66, −0.32). This is particularly relevant to mention as these effects are in the range of improvements in Yo-Yo test performance following 3–8 weeks of sprint training or speed endurance training (Cohen’s *d*: 0.30–0.45) [31,32,33].

In addition to Yo-Yo test performance, this meta-analysis reported that mental fatigue resulted in a lower number of points per shot and shot speed in the Loughborough soccer shooting test. Additionally, mental fatigue negatively affected all of the analyzed outcomes in the Loughborough soccer passing test. Specifically, it seems that mentally fatigued athletes are more prone to making technical errors, as evidenced by the increase in penalty time, which is given for different errors (e.g., missing the target area) [14]. Research has established that mental fatigue increases the attention to irrelevant, compared to relevant stimuli, which might explain poorer technical performance in the Loughborough tests observed herein [34]. Similar to our findings, studies have also reported a lower number of total passes during small-sided games following a mentally fatiguing task [35]. Nevertheless, it remains unclear if these negative effects also translate to performance in sport-specific situations. Some researchers have suggested that the Loughborough soccer passing test might not be a valid test of in-game passing performance, at least among youth players [36]. While this area certainly merits future research, it seems reasonable to suggest that mental fatigue should be avoided before competitions to reduce the likelihood of impairment of technical performance.

There are several limitations of the present review that need to be considered. First, it should be mentioned that all of the included studies involved male participants. Therefore, the results presented herein may not necessarily be generalized to females, and future research in this population is therefore needed. Additionally, the population analyzed among the included studies varied from physically active males to elite athletes (Table 2). The magnitude of the effect observed in most studies was similar, suggesting that the effects of mental fatigue on performance in the three analyzed tests are not population-specific. However, future studies are needed to directly explore the effects of mental fatigue on performance in different populations.

All included studies were classified as being of good methodological quality. Five studies scored 6 points on the PEDro checklist, while two studies scored 8 points. These two studies [4,8] scored more points, given that they also incorporated blinding in their study design. Specifically, these two studies incorporated blinding of assessors measuring the outcomes. Despite the difference in blinding across the included studies, it is interesting to note that generally, all studies reported similar effect sizes with overlapping 95% CIs. This would suggest that the lack of blinding in some studies may not have influenced outcomes.

## 5. Conclusions

This meta-analysis found that mental fatigue negatively impacts different aspects of physical and technical exercise performance. Specifically, mental fatigue negatively impacted endurance-based running, as evident by a lower distance covered in the Yo-Yo test. Furthermore, outcomes such as the original time needed to complete the test, penalty time, performance time, points per shot, and shot speed in the Loughborough soccer tests were negatively affected by mental fatigue. These findings demonstrate that mental fatigue negatively impacts endurance-based running performance as well as soccer passing and shooting skills. Most effects sizes were in the range from small to medium and are likely to be practically relevant.

## Figures and Tables

**Table 1:** Summary of the main outcomes in the three analyzed tests.

Test	Outcome
Yo-Yo test	Distance covered
Loughborough soccer passing test	The original time needed to complete the test Penalty time (i.e., time accounted for errors made during the test) Performance time (i.e., original time plus penalty time)
Loughborough soccer shooting test	Points per shot Shot speed Shot sequence time

**Table 2:** Summary of the studies included in the review.

Study	Participants	Control Task	Cognitive Task	Test and Outcomes
Filipas et al. (2021)	36 male soccer players (*n* = 12 under-14, *n* = 12 under-16, and *n* = 12 under-18)	Reading magazines (15 min)	Computerized Stroop test (30 min)	Yo-Yo IR1—distance covered LSPT—original time, distance covered, and performance time LSST—points per shot, shot speed, and shot sequence time
Greco et al. (2017)	16 young male soccer players ^a^	“normal activities”	Using the “Brain It On” application on a smartphone (30 min)	Yo-Yo IR1—distance covered LSPT—original time, distance covered, and performance time
Lam et al. (2021)	9 physically active males	No activity	Computerized Stroop test (30 min)	Yo-Yo IR1—distance covered
Penna et al. (2018)	12 handball players	Watching an emotionally neutral video (30 min)	Computerized Stroop test (30 min)	Yo-Yo IR1—distance covered
Smith et al. (2016)	12 moderately trained soccer players and 14 experienced soccer Players ^b^	Reading magazines (30 min)	Computerized Stroop test (30 min)	Yo-Yo IR1—distance covered LSPT—original time, distance covered, and performance time LSST—points per shot, shot speed, and shot sequence time
Veness et al. (2017)	10 elite male cricket players	Reading magazines (15 min)	Computerized Stroop test (30 min)	Yo-Yo IR1—distance covered
Weerakkody et al. (2021)	25 male community-level Australian football players	Watching an emotionally neutral video (30 min)	Computerized Stroop test (30 min)	Yo-Yo IR1—distance covered

^a^ 8 participants performed the Yo-Yo test and 8 participants performed the LSPT; ^b^ 12 participants performed the Yo-Yo test and 14 participants performed the LSPT; LSPT: Loughborough soccer passing test; LSST: Loughborough soccer shooting tests; IR1: intermittent recovery level 1.

**Table 3:** Results from the methodological quality assessment using the PEDro checklist.

Reference	Item 1	Item 2	Item 3	Item 4	Item 5	Item 6	Item 7	Item 8	Item 9	Item 10	Item 11	Total Score
Filipas et al. (2021)	Yes	Yes	Unclear	Yes	No	Yes	Yes	Yes	Yes	Yes	Yes	8
Greco et al. (2017)	Yes	Yes	Unclear	Yes	No	No	No	Yes	Yes	Yes	Yes	6
Lam et al. (2021)	Yes	Yes	Unclear	Yes	No	No	No	Yes	Yes	Yes	Yes	6
Penna et al. (2018)	Yes	Yes	Unclear	Yes	No	No	No	Yes	Yes	Yes	Yes	6
Smith et al. (2016)	Yes	Yes	Unclear	Yes	No	Yes	Yes	Yes	Yes	Yes	Yes	8
Veness et al. (2017)	Yes	Yes	Unclear	Yes	No	No	No	Yes	Yes	Yes	Yes	6
Weerakkody et al. (2021)	Yes	Yes	Unclear	Yes	No	No	No	Yes	Yes	Yes	Yes	6

Yes: criterion is satisfied; No: criterion is not satisfied; Unclear: unable to rate.

## Data Availability

Data used for the meta-analysis are available on request from the corresponding author.
